# Air–Ground Collaborative Autonomous Exploration and Mapping Method for Complex Multi-Grain Pile Environments

**DOI:** 10.3390/s26072184

**Published:** 2026-04-01

**Authors:** Lan Wu, Menghao Chen, Xuhui Liang

**Affiliations:** School of Mechanical and Electrical Engineering, Henan University of Technology, Zhengzhou 450001, China; cmh@stu.haut.edu.cn (M.C.); 2024930880@stu.haut.edu.cn (X.L.)

**Keywords:** granary intelligence, autonomous exploration, air–ground collaboration, 3D mapping, task allocation, LiDAR, task allocation

## Abstract

**Highlights:**

**What are the main findings?**

- In this study, we designed a methodology for air–ground co-exploration in a manner that leverages viewpoint complementarity. It also incorporates dust-aware LiDAR fusion for the mapping of complex granary environments.
- Based on our simulation results, system coverage of grain piles reaches 97.2% and exploration time is reduced by 20.1% in contrast to the results obtained with a single robot.

**What are the implications of the main findings?**

- The method enhances the reliability of point clouds generated from uncolored 3D laser scanning. By taking dust-induced deterioration and obstruction into account, the method can ensure high-quality reconstruction, which is essential for the intelligent operation of granaries.
- The described technique presents a viable solution for heterogeneous robot coordination, facilitating autonomous inspection in unstructured, Global Positioning System (GPS)-restricted, and dust-prone industrial sites.

**Abstract:**

Prompt 3D mapping of grain storage is essential for effective management. However, standard mapping algorithms encounter a number of challenges, with the typical granary environment containing dust, grain piles, and narrow aisles. A single robotic agent is not able to provide complete area coverage, and most multi-robot approaches involve re-scanning the same areas due to a lack of explicit viewpoint-based task allocation processes. In order to overcome the above issues, we propose an air–ground collaborative exploration system for complex multi-grain pile scenarios. Exploration redundancy can be reduced by estimating the advantages of viewpoints through ray tracing and assigning the tops of the grain piles to aerial robots with ground vehicles in lower regions and narrow aisles. In order to manage dense dust (5–15 mg/m^3^), the quality-aware fusion strategy evaluates the reliability of the distance and point density of the sensing to reduce the influence of degraded aerial depth data. Moreover, mapping relies on LiDAR data to ensure mapping quality. A mechanism for re-scanning to enable coverage-driven exploitation of insufficiently explored regions is subsequently proposed. The simulation results show that the design achieved a grain pile coverage of 97.2%, with the total exploration time reduced by 20.1% over single-robot baselines. The results indicate that viewpoint-aware task allocation and dust-sensitive perception fusion can offer a practical solution for autonomous inspection in GPS-restricted, dust-rich industrial environments, such as granary facilities.

## 1. Introduction

With the continuous implementation of the national food security policies, granary facilities are being equipped with intelligent management systems gradually [1]. In these modern facilities, an increasing number of autonomous robots are deployed for various downstream missions. Among these, grain leveling is a vital but fragile and labor-intensive task. With the complete 3D maps generated by our proposed collaborative system, geometric prior information can be provided to allocate tasks globally, avoid obstacles, and plan safe paths for heavy grain-leveling robots. In addition, the system provides accurate spatial representations for localized fumigation, anomaly detection, pest biocontrol, and inspection tasks. Therefore, high-fidelity 3D surface reconstruction is necessary for reliable grain volume estimation and rapid inventorying [2]. Autonomous 3D mapping of granary interiors is thus not an isolated perception task, but a key enabler for downstream agricultural and management interventions [2,3]. Recent studies [4] suggest that operational accuracy can be achieved only with dense and complete point clouds. Nonetheless, manual measurements are labor-intensive and may be unsafe. Meanwhile, fixed sensor networks also lack the flexibility to adapt to dynamically changing grain pile distributions [5]. The interior area of a granary is typically rich in airborne dust, potentially ranging from 5 to 15 mg/m^3^. Significant variations in floor and pile heights, combined with narrow working passages between grain piles, present significant challenges to autonomous exploration systems in confined or cluttered spaces [6]. Due to visual occlusion and the limited field of view of aerial platforms, perception remains inefficient in lower regions and narrow aisles, observations based on recent Unmanned Aerial Vehicle (UAV)-based exploration studies [7]. The performance of depth sensing, in particular, decreases significantly in dusty conditions [8]. Ground vehicles are constrained by limitations because of restrictions imposed by mounting height and the viewpoint of sensors, creating persistent blind spots on grain pile tops [9]. Consequently, single-platform viewing provides limited and unreliable coverage in granaries.

To increase exploration efficiency and coverage, multi-robot collaborative exploration has been investigated extensively [10,11,12,13,14,15]. The communication overhead of heterogeneous robot teams is lower than that of homogeneous robots. Thus, the former can simplify exploration tasks in a cost-effective manner [16]. Heterogeneous systems, particularly air–ground collaboration, represent a more pragmatic solution given their complementary sensing perspectives [17,18,19]. To date, studies have been conducted on air–ground cooperation of hybrid mobility platforms [20]; aerial-assisted ground navigation [21]; and integrated aerial–ground robotic systems for agriculture [22]. This partnership has been further transferred to mapping and exploration in GPS-restricted environments [23,24,25,26,27]. Zheng et al. recently proposed an air-assisted ground exploration framework for large-scale unknown environments [28], and Xu et al. developed a collaborative Simultaneous Localization and Mapping (SLAM) system that explicitly fuses aerial and terrestrial LiDAR data [29].

While UAV-Unmanned Ground Vehicle (UGV) collaboration has been shown to be useful for outdoor agricultural applications, such as targeted spraying in orchards [30], adopting these frameworks in indoor granary settings is not straightforward. Granaries present a different set of interrelated challenges that are not captured in previous benchmarks. These include severe vertical occlusions due to the steep geometry of grain piles and narrow aisles, as well as dense airborne dust (typically 5–15 mg/m^3^) that degrades optical depth sensing performance [31]. Overall, autonomous exploration that addresses these interconnected problems remains limited, despite advances in related fields. When deployed in these settings, existing state-of-the-art collaborative exploration methods—which primarily allocate frontier regions (formally defined as the boundary zones separating known free space from unexplored areas) using distance- or cost-based criteria [32]—often result in redundant cross-platform observations and poor exploration efficiency in vertically occluded spaces [33]. In addition, fusion pipelines that assume stable sensor performance often accumulate low-quality depth measurements, resulting in incomplete and noisy reconstructions [8,34]. These distinct hurdles indicate that there is a clear need for the proposed approach. Instead of relying on distance alone to assign the two types of robots, platform-specific information gain is used to compute viewpoint complementarity. Exploration tasks are decoupled during robot assignment to improve allocation efficiency: UAVs are tasked with covering grain pile tops, while UGVs concentrate on lower regions and narrow passages. The core distinctions between our method and existing frameworks are summarized in Table 1.

When conducting simulations under realistic granary conditions, it is observed that the proposed framework achieves higher coverage and lower exploration redundancy than comparative single-robot and distance-based collaborative approaches. Compared to baseline methods, our 3D reconstruction method produces significantly more complete reconstructions while avoiding redundant observations in occluded and dusty regions. The results indicate that the proposed method of viewpoint-aware task allocation combined with dust-sensitive perception fusion can feasibly achieve high-fidelity 3D mapping in dusty, GPS-restricted environments, specifically grain silos [39,40].

We present a dust-sensitive multi-sensor fusion approach that takes into account spatial information of observation distance, incidence angle and local point density. This formulation allows the system to down-weight unreliable measurements in dusty zones instead of ignoring them completely. We also present a mechanism for coverage-driven re-scanning to identify and re-scan uncovered areas. While designed specifically for grain storage buildings, these design decisions will nonetheless be effective in other indoor or industrial settings that involve dust, occlusion, and multiple levels.

In order to realize an actual operation state, the simulator needs to follow a close-to-reality working process in practice. In the idealized simulation, collaborative frontier estimation usually assumes perfect inter-robot Communication and fully synchronized map update. In practice, due to limited physical conditions such as communication latency across grains and signals lost in them; As well as an asynchronous deviation in state estimation.

## 2. Materials and Methods

### 2.1. System Architecture and Workflow

**Figure 1:**
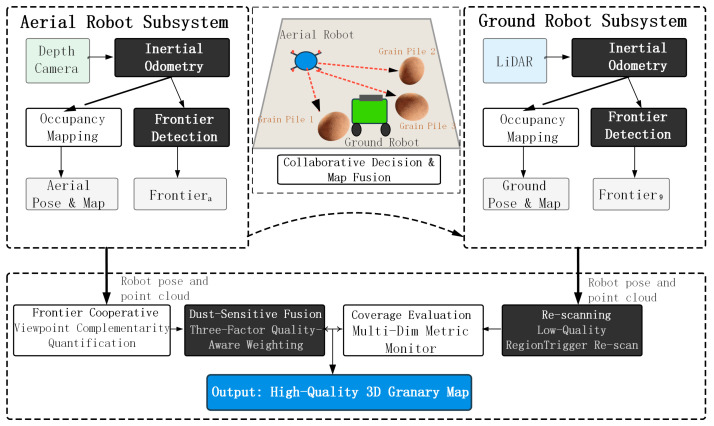
Framework of air-ground collaborative autonomous exploration and mapping system for granary environments.

The air-ground collaborative system developed in this study was based on an asynchronous parallel architecture. In established serial frameworks, robots must wait for command updates; in comparison, UAVs and UGVs execute exploration and mapping tasks concurrently. As seen in Figure 1, the architecture manages three modules: the Aerial Robot Subsystem, the Ground Robot Subsystem, and the Collaborative Decision Module.

As shown in Figure 1, the complete system architecture can be logically divided into three main parts: two independent robot perception subsystems and a collaborative decision and map fusion module.

The Aerial Robot Subsystem uses a depth camera fused with inertial odometry to capture the surface shape of high-level grain piles. This subsystem independently generates an aerial occupancy map and performs local frontier detection, referred to as ${Frontier}_{a}$. At the same time, the Ground Robot Subsystem utilizes a LiDAR sensor together with inertial odometry to map lower zones and narrow aisles. It creates its own ground pose estimation, local map, as well as detected frontiers, referred to as ${Frontier}_{g}$.

This means that the data streams from both subsystems—including robot poses, point clouds, and local frontier results—are sent to the Collaborative Decision and Map Fusion module, represented by the large dashed frame in the bottom of Figure 1. Inside this centralized module, the Frontier Cooperative Allocation unit first computes viewpoint complementarity for task assignment: the UAV will focus on the tops of the grain piles, while the UGV will cover the lower regions.

Subsequently, the Dust Sensitive Fusion unit employs a three-factor quality-based weighting rule to proactively filter noise from depth measurements that are caused by dust. Also, it adaptively merges the aerial and ground point clouds. The Coverage Evaluation unit continuously tracks the completeness of maps using diverse metrics. When map quality falls below a threshold, the Re-scanning unit is activated to guide the robots to low-quality areas. In this closed-loop manner, the system constructs and progressively refines a high-quality 3D granary map with consistent global alignment.

#### 2.1.1. Aerial Robot Subsystem

Aerial Robot Subsystem: Based on a quadrotor flight platform, it is equipped with a depth camera and an Inertial Measurement Unit (IMU). For autonomous self-localization, we use Visual-Inertial System (VINS)-Fusion [41], which achieves an approximate position error within 0.05 m and performs robustly in our scenarios. VINS-Fusion is a vision–inertial state estimator based on nonlinear optimization. Its primary idea is to maintain a sliding window of recent keyframes and jointly optimize the full system state. The estimated state includes the robot’s position, velocity, and IMU biases. Convergence is achieved by minimizing a single nonlinear cost function that comprises a prior term from marginalization, residuals from IMU pre-integration, and visual reprojection errors. This graph-based optimization framework provides accurate six degrees of freedom (6-DoF) odometry. Stable pose estimation is essential for the UAV to fly safely and to navigate steadily above grain piles with an uneven and continuously changing surface. To overcome the problem of signal fading in dense particulate air, the system enforces a strict near-range limit (<3 m) [42]. Restricting observations to this near-field zone maintains high depth accuracy (±10 mm) and avoids dust. With this configuration, the UAV is tasked with obtaining macroscopic structural data and covering the otherwise inaccessible wild grain piles.

Depth image projection transformation is defined as follows:

(1) $$P^{C}=d\left(u,v\right)\cdot K^{-1}\cdot \left[\begin{array}{c} u\\ v\\ 1 \end{array}\right]\in R^{3}\;P^{W}=T_{C}^{W}\cdot \left[\begin{array}{c} P^{C}\\ 1 \end{array}\right]=\left[\begin{array}{cc} R_{C}^{W} & p_{C}^{W}\\ 0^{T} & 1 \end{array}\right]\left[\begin{array}{c} P^{C}\\ 1 \end{array}\right]\in R^{3}$$

In Equation (1), (u,v) denotes the 2D pixel coordinates and d(u,v) is the corresponding metric depth. The camera intrinsic matrix $K\in R^{3\times 3}$ defines the optical projection model, mapping 2D pixels to 3D directional rays that generate a local point $P^{C}\in R^{3}$. The transformation from the camera frame (C) to the global world frame (W) is given by $T_{C}^{W}\in SE(3)$. The rotation matrix $R_{C}^{W}\in SE(3)$ and translation vector $p_{C}^{W}\in R^{3}$ are continuously updated by visual–inertial odometry and are used to project local geometric features ($P^{C}$) into the global coordinate frame ($P^{W}$).

The system processes 5000–8000 valid depth points per frame in 25–35 ms. However, these measurements become unreliable in cluttered and dusty environments. Following prior studies, the empirical depth error increases from ±10 mm under clean conditions to approximately ±80 mm under dusty conditions (i.e., beyond 5 m). This degradation was quantified through systematic comparisons between the depth camera measurements and a high-resolution laser rangefinder, which served as ground truth on a flat calibration surface.

#### 2.1.2. Ground Robot Subsystem

The ground unit is built on a differential-drive chassis equipped with a 16-line LiDAR and an IMU. LIO-SAM [43] is used to provide reliable and accurate state estimation in granary aisles with limited geometric features. As a tightly coupled LiDAR–inertial framework, LIO-SAM formulates state estimation as a factor graph optimization problem, integrating IMU pre-integration with LiDAR feature matching to achieve real-time trajectory estimation. The reported localization accuracy of ±0.03 m (corresponding to a 0.25% relative error) is supported by benchmark results from Shan et al. [43]. The UGV is then responsible for mapping the bottom and sides of grain piles and the narrow passages (0.6–1.2 m). A practical advantage is that LiDAR, unlike optical sensors, is largely insensitive to airborne dust particles at concentrations of 5–15 mg/m^3^ and can maintain an accuracy of ±20 mm even under poor air quality [5].

The ground robot adopts factor graph optimization for pose estimation. The optimization objective is as follows:

(2) $$X*=arg\;\underset{X\in X}{min}\;\sum_{f_{i}\in F}\;\rho (∥r_{i}(X_{i}){∥}_{\Sigma_{i}}^{2})\;$$

subject to

(3) $$\left\{\begin{array}{c} r_{edge}(X)=\frac{∥(p-q)\times d∥}{∥d∥},\;\;\;\;Edge\;constraint\\ r_{surf}(X)=\frac{\left|n^{T}\cdot p-c\right|}{∥n∥},\;\;\;\;\;\;\;\;Planar\;constraint \end{array}\right.\;$$

where $X=\{X_{1},\ldots ,X_{n}\}\in X$ is the set of state variables (pose, velocity, IMU bias); $r_{i}:X\to R^{n}$ is the residual mapping; $∥\cdot {∥}_{\Sigma_{i}}^{2}$ is the Mahalanobis distance; ρ:R→R is the Huber robust kernel function; p, q are edge points; d is the edge direction; n is the plane normal vector; and c is the plane offset. The optimization time is 18–25 ms/frame.

#### 2.1.3. Collaborative Decision Module

The Collaborative Decision Module serves as the centralized control center of the multi-robot architecture. Mission execution is coordinated with operational safety and collision avoidance as primary priorities. Real-time pose and point-cloud data from both the UAV and UGV are processed to support four coordinated sub-processes: cooperative frontier allocation, dust-sensitive map fusion, coverage evaluation, and targeted re-scanning.

Instead of relying on standard distance-based heuristics, the task-allocation mechanism computes viewpoint complementarity. The UAV is assigned to explore high-elevation grain surfaces, while the UGV maps lower aisles, improving vertical coverage and reducing redundant cross-platform observations. The map-fusion unit evaluates dust-degraded visual measurements and down-weights them to preserve the fidelity of the global 3D reconstruction.

Mapping progress is monitored using multi-dimensional metrics. The mission follows strict termination criteria and ends only when the global exploration quality metric ($Q_{explore}$) exceeds 0.90 and the total geometric coverage of grain piles surpasses 95%. This centralized design exploits complementary sensing modalities across heterogeneous platforms to enable robust exploration in confined, dust-prone environments.

### 2.2. Probabilistic Environmental Representation

We employ a probabilistic Occupancy Grid Map (OGM) for environmental modelling [44]. The method divides the 3D exploration space into a regular voxel, where each type of voxel has an occupancy probability. The system employs progressive modeling rather than snapshot modeling, continuously fusing incoming multisensory observations via Bayesian filtering to update the state of each voxel in real time.

#### 2.2.1. Map Discretization and Log-Odds Representation

The mapping framework establishes the foundation by discretizing the 3D workspace Ω $=[x_{min},x_{max}]\times [y_{min},y_{max}]\times [z_{min},z_{max}]\subset R^{3}$ into a voxel grid, whose resolution, r, acts as the main trade-off variable. In order to save computing resources, we assign a finer detail resolution ($r_{a}=0.1$ m) to the aerial robot compared to the ground robot ($r_{g}=0.2$ m). The above dimensions of the granary, 20×20×3 m, yield manageable voxel counts of $1.2\times 10^{6}$ for the aerial map and $1.5\times 10^{5}$ for the ground map, thus enabling them to be used in resource-constrained embedded platforms [36].

Each voxel $v_{i}\in V$ maintains its occupancy state. The function L:V→R con maps the occupancy probability $P(v_{i})$ to a numerically stable logit form:

(4) $$L\left(v_{i}\right)=logit\left(P\left(v_{i}\right)\right)=log\left(\frac{P\left(v_{i}\right)}{1-P\left(v_{i}\right)}\right)\;$$

#### 2.2.2. 3D-DDA Ray Tracing and Bayesian Update

For real-time operation, the 3D-Digital Differential Analyzer (3D-DDA) algorithm is utilized for ray tracing. The ray parameterization and step increment are described and can be written as follows:

(5) $$\begin{array}{c} r(t)=p_{s}+t\cdot (p_{o}-p_{s}),t\in [0,1]\\ t_{\Delta x}=\frac{sgn(d_{x})}{d_{x}},t_{maxx}^{\left(k+1\right)}=t_{maxx}^{\left(k\right)}+t_{\Delta x} \end{array}\;$$

The sensor origin and obstacle point are referred to as $p_{s}$ and $p_{o}$, respectively. The algorithm traverses the voxel sequence $V_{ray}=\{v_{1},v_{2},\ldots ,v_{n}\}$ until ray termination.

The recursive Bayesian rule is used to update the state of the voxel, enabling the map to be incrementally constructed:

(6) $$\left\{\begin{array}{c} L(v_{i}|z_{\left(1:t\right)})=L(v_{i}|z_{t})+L(v_{i}|z_{\left(1:t-1\right)})-L(v_{i}|\phi )\\ L_{new}(v_{i})=clamp(L_{old}(v_{i})+L_{miss},L_{min},L_{max}),\;\;\;\;v_{i}\in V_{free}\\ L_{new}(v_{i})=clamp(L_{old}(v_{i})+L_{hit},L_{min},L_{max}),\;\;\;\;\;\;v_{i}=v_{end} \end{array}\right.$$

The parameters were selected to achieve robust performance across different sensors. Specifically, the hit increment $L_{hit}\approx 0.847$ and miss decrement $L_{miss}\approx -0.619$ were set within a clamping range of [−1.988,3.476] at an occupancy threshold of $L_{occ}=1.386$.

To avoid redundant computations, we used a flag mechanism (flag_traverse, the flag_rayend) that stores the ray ID (raycast_num) when updating a voxel. It is important to ensure that the same voxel data are not accessed multiple times by the same ray. As a result, computation time is reduced from 150 ms to 30 ms, an 80% acceleration, enabling stable 10 Hz real-time operation (32 ms on aerial and 28 ms on ground robots).

### 2.3. Viewpoint Complementarity and Collaborative Exploration Strategy

To enable efficient exploration, it is essential to define the boundaries between known and unknown space. These limits are known as the frontiers. In the case of granaries, all of these frontiers generally cluster at the top, bottom, and sides of the grain piles. In narrow aisles, the limits also cluster.

#### 2.3.1. Frontier Detection and Information Gain Calculation

The candidate front set C represents the free voxels adjacent to the unknown space, which are considered for exploration [6]:

(7) $$C=\{v\in V|occ(v)=free\wedge \exists v^{\prime }\in N_{4}(v):occ(v^{\prime })=unknown\}\;$$

Here, $N_{4}(v)$ denotes the 4-neighborhood (planar) to reduce vertical noise.

A localized Breadth-First Search (BFS) is conducted (rather than a global search), commencing from the robot’s position $p_{robot}$ and with a search radius of $R_{search}$ (15 m for aerial, 20 m for ground). Using a clustered approach, points close together (<0.4 m) are filtered and joined together via a disjoint-set structure. Any insignificant structures (<20 voxels) are then discarded. For each valid cluster $f_{k}$, the centroid $p_{k}^{c}$ and normal vector $n_{k}$ are as follows:

(8) $$p_{k}^{c}=\frac{1}{\left|{\mathrm{cluster}}_{k}\right|}\sum_{v_{i}\in {\mathrm{cluster}}_{k}}\;pos(v_{i})\in R^{3},n_{k}=PCA({\mathrm{cluster}}_{k})\in R^{3}\;$$

**Figure 2:**
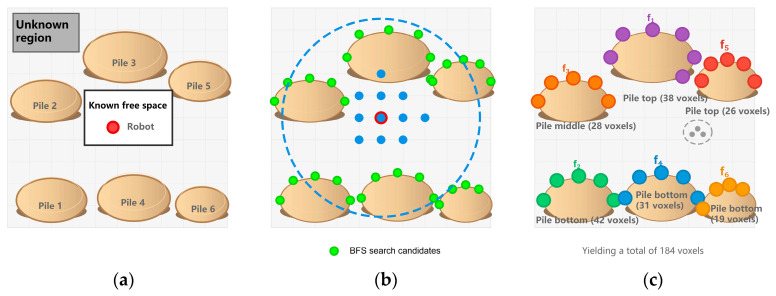
Frontier detection and clustering within the grain-storage environment: (**a**) Occupancy map; (**b**) BFS search trajectory, where the dashed line indicates the search range and the blue points denote the traversed grid points; (**c**) Clustering results highlighting the pile tops and bottoms.

The local BFS strategy drastically reduces detection time from one hundred to one hundred and fifty milliseconds to six to twelve milliseconds, a 10–13 fold improvement over global methods. As seen in Figure 2, it is effective to differentiate the top and bottom of grain piles, which enables a complementary air–ground survey.

The observation location and information feedback for robot j∈{a,g} are defined as follows:

(9) $$\begin{array}{c} p_{view}^{j}(f_{k})=\left\{\begin{array}{cc} p_{k}^{c}+h_{a}\cdot n_{k}, & j=a\;(h_{a}=2\;m)\\ p_{k}^{c}, & j=g \end{array}\right.\\ \;\;\;\;\;\;\;\;\;\;\;\;\;\;\;\;\;\;\;\;\;\;\;\;\;\;\;\;\;\;\;\;\;\;\;\;\;\;\;\;\;\;\;\;\;I_{j}(f_{k})=\left|\underset{{\mathrm{ray}}_{i}\in R_{j}}{⋃}\;\left\{v\in V_{ray}({\mathrm{ray}}_{i})|occ(v)=unknown\right\}\right|\in Z^{+} \end{array}\;$$

The statement *pviewj* is a position of observation; *Rj* is the set of rays sampled along the element view frustum from the observation position; $I_{j}:F\to Z^{+}$ is the information gain mapping from the union of unknown voxels visible to all rays. Aerial depth camera: horizontal 87°, vertical 58°, sampling interval 5°, $\left|R_{j}^{a}\right|$ number of rays $|R_{j}^{a}|\approx 180$, LiDAR for horizontal 360° vertical ±15° with a sampling interval of 10°, and number of rays $|R_{j}^{g}|\approx 576$. The information gain is cached by the system and re-calculated only when the map changes (new voxels > 100) or when the Frontier moves (>0.5 m). The calculation completion time for the aerial is 15 ms; in comparison, that of the ground is 8 ms. Aerial advantage yields $I_{a}=2500$ versus $I_{g}=200$ for grain pile tops; in comparison, ground advantage yields $I_{g}=3200$ versus $I_{a}=600$ for grain pile bottoms/passages. The granaries are the best environment for testing heterogeneous cooperation as strata are structurally complementary.

It can be noted that in the current simulation framework, the collaborative frontier estimation depends on the stable pose graphs and unconstrained bandwidth. When we emulate something physically, we will have challenges due to real-world sensor noise and asynchronous transmission when extracting and sharing these frontiers.

#### 2.3.2. Viewpoint Complementarity Quantification and Collaborative Allocation

**Figure 3:**
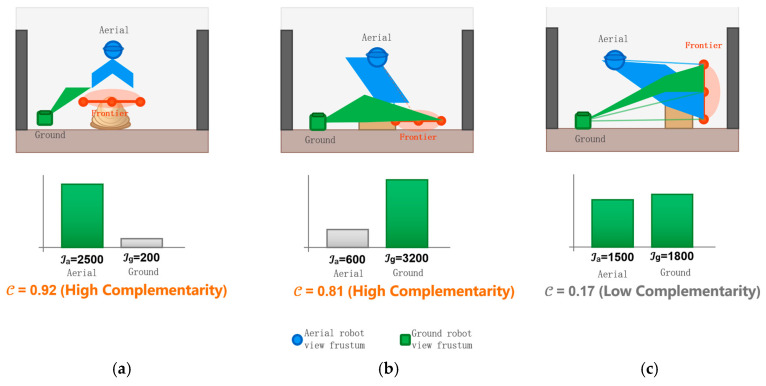
Comparison of air-ground information gain: (**a**) Pile-top frontiers (>2.5 m): the UAV view frustum covers the target, yielding high $I_{a}$. (**b**) Bottom and passage frontiers (<0.8 m): the UGV has a clear line of sight, yielding high $I_{g}$. (**c**) Middle frontiers (0.8–2.5 m): fields of view overlap, producing similar gains. Bars show $I_{a}$ and $I_{g}$; $C(f_{i})$ is the viewpoint complementarity.

To allow for a quantitative justification of the proposed collaborative allocation strategy, Figure 3 displays the structural difference in view coverage of the heterogeneous platforms. As displayed in Figure 3a, a frontier located at the top of the grain piles with a height greater than 2.5 m offers an information gain to the aerial sensor ($I_{a}\approx 2500$) that is substantially more than that of the heavily occluded ground sensor ($I_{g}\approx 200$). In contrast, Figure 3b shows that base regions and narrow passages (height < 0.8 m) strongly promote the ground robot viewpoint ($I_{g}\approx 3200$ vs. $I_{a}\approx 600$). The view frustums for both platforms overlap in transitional middle layers (0.8–2.5 m, Figure 3c), contributing similar and redundant information gains. ($I_{a}\approx 1500$, $I_{g}\approx 1800$).

The difference in information gain allows us to formalize this observation into a computable quantity: viewpoint complementarity $C(f_{i})$.

The equation is specified as follows:

(10) $$C(f_{i})=\frac{\left|I_{a}\left(f_{i}\right)-I_{g}\left(f_{i}\right)\right|}{\max\left(I_{a}\left(f_{i}\right),I_{g}\left(f_{i}\right)\right)}\in [0,1]\;$$

Here, C→1 indicates a strong preference for a platform (e.g., pile tops at C=0.92), whereas C→0 indicates ambiguous regions (e.g., middle layers at C=0.17). This metric is the main filter for our allocation logic.

The task assignment is based on a utility function defined over four competing factors: raw information gain, complementarity alignment, travel cost, and overlap penalty:

(11) $$U_{ij}=w_{G}\cdot \frac{max(I_{a}(f_{i}),I_{g}(f_{i}))}{I_{max}}+w_{C}\cdot C_{signed}(f_{i},r_{j})-w_{D}\cdot \frac{∥p_{j}-p_{i}^{c}∥}{d_{max}}-w_{O}\cdot P_{overlap}(f_{i})$$

Weight settings ($w_{G}=0.4$, $w_{C}=0.3$, $w_{D}=0.2$, $w_{O}=0.1$) are designed to enable efficient exploration, assigning a very high reward to the correct robot–task pair with the signed complementarity factor $C_{signed}$. As a result, frontiers with high complementarity (C>0.7) are almost entirely assigned to the advantaged robot enforcing the “air-for-tops, ground-for-bottoms” strategy.

#### 2.3.3. Path Planning for Air–Ground Robots

Upon receiving the target assignment, the system generates the collision-free trajectories based on the kinematic constraints of the individual robots. The aerial planning module uses A* on the 3D grid map with a self-defined cost function: f(n)=g(n)+h(n)

(12) $$\begin{array}{c} g(n)=\sum_{i=1}^{n-1}\;∥p_{i}-p_{i+1}∥+\lambda_{alt}\cdot \Delta z\\ h(n)=∥p_{n}-p^{goal}∥ \end{array}\;$$

Importantly, the cost function has a height penalty $\lambda_{alt}=0.3$, which discourages every unnecessary change in altitude, stabilizing the flight within the corridor ($1.5\leq z_{i}\leq 6.5$ m) of safety. The generated raw path is smoothed by using B-spline curves to satisfy dynamic limits ($v_{max}=1.5$ m/s, $a_{max}=1.2$ m/s^2^).

The ground robot with a 2-layer architecture maneuvers through tight grain pile aisles (0.6–1.2 m width). The global guidance is implemented via A*, whereas the local reactive control handles obstacle avoidance through DWA (Dynamic Window Approach). The DWA objective function aims to optimize three different terms:

(13) score(v,ω)=α⋅heading(v,ω)+β⋅dist(v,ω)+γ⋅velocity(v,ω)

Parameter tuning favors goal alignment (α=0.4, β=0.4) and safety over raw speed (γ=0.2). This integrated pipeline works incredibly well: it reduces redundant exploration from 24.7% (baseline) to 2.8% and achieves 93.5% correct assignments for aerial tasks on pile tops.

### 2.4. Dust-Sensitive Fusion and Quality Assurance

Allocation of observing time on the basis of efficiency solves the “where to look” problem. However, the “how to see” problem is often critical and significantly affected by dust (5–15 mg/m^3^). The proposed dual-layer solution of a quality-aware fusion strategy to filter noise and a closed-loop re-scanning mechanism for missed areas is detailed in this section.

#### 2.4.1. Dust-Sensitive Multi-Sensor Fusion

The quality model Q(p) is grounded in the physical behavior of active optical sensors in particulate-laden environments. Airborne dust induces strong Mie scattering, causing exponential attenuation of the emitted light signal with increasing distance [8]. In addition, according to Lambert’s cosine law, the reflected intensity decreases substantially at large incidence angles. Finally, dust particles typically generate sparse and isolated point clusters, which are structurally distinct from the dense returns of solid grain surfaces. Based on these physical considerations, a three-factor quality model Q(p) is adopted to down-weight unreliable points:

(14) $$Q(p)=q_{d}(p)\cdot q_{\theta }(p)\cdot q_{\rho }(p)\in [0,1]$$

Distance Factor $q_{d}(p)$: The dust effect of a depth camera is modeled with an exponential decay for distances bigger than 3 m. As shown in Equation (15), after 5 m, the distance factor drops dramatically, but LiDAR remains stable ($q_{d}\equiv 1.0$).

Distance Factor $q_{d}(p)$: Dust covers the projector light, meaning that objects at distances greater than 5 m are not influenced by the projector light. We therefore apply exponential decay for depth cameras:

(15) $$\left\{\begin{array}{c} 1.0,\;\;\;\;\;\;\;\;\;\;\;\;\;\;\;\;\;\;\;d<3\;m\;(high\;quality)\\ \exp(-(d-3)/2),\;\;\;\;3\leq d\leq 8\;m\;(gradual\;decay)\\ 0.1,\;\;\;\;\;\;\;\;\;\;\;\;\;\;\;\;\;\;\;d>8\;m\;(low\;quality) \end{array}\right.$$

where $d=\;∥p-p_{sensor}∥$ is the observation distance. For LiDAR, $q_{d}$ is 1.0 (stable in high dust). The distance thresholds in Equation (15) were determined through preliminary empirical calibration. The depth camera was tested in a controlled enclosed environment with a maximum dust concentration of 15 mg/m^3^. Statistical analysis showed that the depth root-mean-square error (RMSE) remained within an acceptable range (<20 mm) for distances up to 3 m. The error then increased rapidly with distance and became unusable (RMSE > 80 mm) beyond 8 m. These values were selected as the lower and upper thresholds for the distance-based confidence decay.

(16) $$q_{\theta }(p)=max(0.1,cos\theta )$$

where θ is the angle between the observation direction and surface normal. This factor reflects the signal loss described by Lambert’s cosine law.

Density Factor $q_{\rho }(p)$: This factor reflects local point cloud density:

(17) $$q_{\rho }(p)=min(1.0,\rho_{local}(p)/\rho_{ref})$$

The notation $\rho_{local}(p)=|\{p^{\prime }|∥p^{\prime }-p∥<r_{\rho }\}|$ denotes the number of points within radius $r_{\rho }=0.5$ m, whereas $\rho_{ref}=50$ is the reference density. These density parameters were calibrated to filter out sparse outlier points generated by airborne dust reflections.

The angle of incidence $q_{\theta }(p)$ and the density $q_{\rho }(p)$ further exacerbate incorrect views and sparse outliers. Fusion is then performed adaptively:

(18) $$w_{a}=\frac{Q_{a}}{Q_{a}+Q_{g}},w_{g}=1-w_{a}\;$$

In the majority of cases, a dust level of 15 mg/m^3^ results in a value of 0.3 for aerial depth confidence ($Q_{a}$) at long range but 0.9 for LiDAR ($Q_{g}$). The fusion engine automatically transfers the trust weight to the LiDAR ($w_{g}=0.75$), preventing noise from the depth camera from corrupting the map.

#### 2.4.2. Quality-Driven Re-Scanning Strategy

To ensure the completeness of the map, a monitoring loop is applied, which is based on a composite quality measure:

(19) $$Q_{explore}(t)=\alpha_{1}\eta_{cov}(t)+\alpha_{2}\eta_{comp}(t)+\alpha_{3}\eta_{util}(t)$$

The term $Q_{explore}(t)$ refers to the exploratory quality measure of the system at time t, whereas the alpha parameters refer to adjustable weighting measures for the respective quality measures. Furthermore, the ($\eta_{cov}$) term is the covariance measure, which focuses on the influencing parameters used to model the system. The ($\eta_{comp}$) is the competitiveness measure that aims to measure the positioning of the dominating players in the market. Lastly, the $\eta_{util}(t)$ is the utility measure, which aims to extract the useful measures for the players themselves. In summary, the presence of the weighting factors helps to control their corresponding impact on the system. Therefore, their values can be adjusted to suit the actor systems as desired. Moreover, the measure of these parameters must be mapped in real time to achieve more effective results.

The three indicators used to measure performance are coverage ($\eta_{cov}$), geometric completeness ($\eta_{comp}$), and complementarity utilization ($\eta_{util}$). Moreover, if the score falls below 0.9, a ‘virtual Frontier’ is created to re-scan the regions.

(20) $$f_{rescan}=(p_{rescan},\;n_{interior},\;priority=HIGH,\;gain=1.5|R|)$$

Using the 1.5× utility multiplier, virtual Frontiers are added to the global task list with high priority. This logic ensures that the system will automatically return to the grain pile to correct any deficiencies. This two-pronged quality assurance using adaptive fusion and targeted re-scanning optimizes local fidelity and global completeness, filling a crucial void commonly ignored within past collaborative mapping research, in that it assesses not coverage speed but data quality. In heavy dust conditions (5–15 mg/m^3^), the mechanism will raise map completeness from a baseline of 76% to 94.3%, while concurrently reducing volumetric error from ±4.2% to ±2.5% (validation in Section 3).

### 2.5. Statistical Analysis

All simulation conditions were statistically verified rigorously in this study to evaluate the effectiveness and reliability of the proposed collaborative exploration system. For each Scenario (simple, large and dense granaries), 10 independent exploration trials of the proposed method and the two baselines, TARE and FAEL, were conducted to eliminate random interference from sensor noise and path-planning errors. Record both the overall completion time of each trial and the final grain pile coverage. The results are presented as the mean ± standard deviation (SD). The above description means that these data present obvious increasing features; The impact from outliers is limited at this time.

## 3. Results

The MARSIM simulation platform was used to validate the air–ground concept proposed and employed to evaluate its performance under realistic constraints. In the following section, we will discuss the experimental setup, present qualitative trajectory comparisons, and deliver a detailed quantitative performance analysis.

### 3.1. Experimental Setup

**Figure 4:**
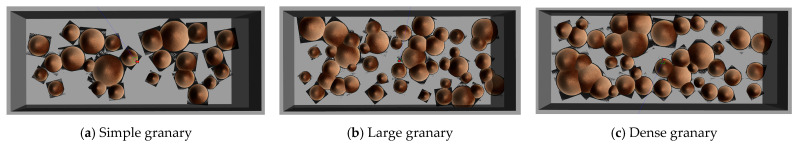
Scenario simulation environments.

The testing environment is based on the MARSIM platform Robot Operating System (ROS) Noetic, which includes a high-fidelity implementation of the Hector quadrotor UAV and the Husky ground robot. The experimental protocols involve the same multi-robot exploration protocols of the CERBERUS system [37,38], which was a successful system in the SubT Challenge [20], thus ensuring that the evaluation protocol involved state-of-the-art techniques. The verification involves three different granary scenarios, with the parameters provided in Table 2 and their layouts presented in Figure 4. To ensure physical realism, the grain piles have non-uniform heights (1.37–3.44 m) and the base radius is height-dependent (1.15–1.59 m). Most importantly, these geometries conform to the actual angle of repose in real grain piles (30°) and pose valid occlusion challenges. Testing consisted of 10 independent trials for each scenario to account for probabilistic variations. All recorded runs had a strict termination criterion: we continued exploration, provided the overall quality score satisfied $Q_{explore}\geq 0.9$, and over 95% of the grain pile was covered, or the maximum timeout of 600 s was reached

The comparative performance evaluation was conducted against two methods: TARE, which is a hierarchical global-local planning framework developed by CMU [35], and FAEL, which is a rapid exploration method from Sun Yat-sen University [36] that is optimized for speed with a heuristic. A significant difference in structure is noted: both TARE and FAEL function only with one UGV platform. Without an aerial view, these baselines have blind spots at the top of any grain piles. The suggested collaborative framework, in contrast, utilizes viewpoint complementarity; the assigning logic signifies that the UAV will map inaccessible tops while the UGV will secure ground aisles.

The estimation and parameterization of dust concentrations (5–15 mg/m^3^) used in the simulation scenarios are not arbitrary. The corresponding values were obtained through empirical spot testing conducted during initial fieldwork in an active flat-warehouse granary, as detailed in Section 3.6. Calibrated portable laser particle counters were deployed at typical UAV operating heights (1.5–3 m) to measure ambient dust concentrations. Under well-ventilated conditions during routine operations, the average background dust concentration can reach 5–15 mg/m^3^ due to grain deposition and localized dusty vortices. These measurements were collected under conditions comparable to those in enclosed, large-scale grain warehouses. Using this validated range as the basis for modeling sensor degradation in the MARSIM platform better reflects real industrial granary environments.

### 3.2. Qualitative Results: Trajectory Comparison

**Figure 5:**
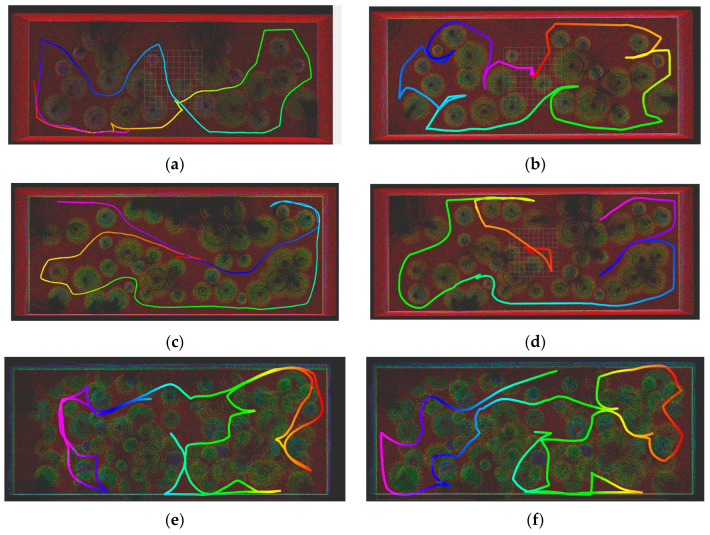
Comparison of UGV Trajectories between the TARE Algorithm and the Proposed Method. (**a**) TARE in the 25-pile scenario; (**b**) Proposed method in the 25-pile scenario; (**c**) TARE in the 40-pile scenario; (**d**) Proposed method in the 40-pile scenario; (**e**) TARE in the 45-pile scenario; (**f**) Proposed method in the 45-pile scenario.

The exploration trajectories, as illustrated in Figure 5, show the single-UGV baseline (TARE) versus the air–ground collaboration for different density levels.

The flaws of a ground-only perspective become immediately clear from (a) and (b) presented in (25), the simple granary example. The TARE trajectory (a) does not map the tops of piles, which results in 87.2% coverage. In comparison, the collaborative system (b) activates the UAV to fill in those blind spots, boosting coverage to 96.8%, a relative improvement of 11.0%.

As complexity increases, individual robots become less efficient. TARE (c) experiences difficulties in negotiating deep, narrow passages (891.50). The proposed method involves the use of complementarity to overcome these limitations, achieving 97.2% coverage (+8.6%).

The 45-pile dense granary scenario was used as the ultimate test case. The single UGV (e) is unable to detect dense peaks, resulting in large map voids and a mere 85.8% coverage. The collaborative approach assures total reconstruction of bottoms, middles, and tops at 97.5% (f), a significant performance increase of 13.6%.

Based on the images obtained, aerial views are not merely ‘good to have’ but essential. The cooperative solution consistently surpasses the 95% coverage threshold established for smart inventory management. Indeed, terrestrial-only systems do not and cannot reach such coverage.

### 3.3. Quantitative Analysis: Performance Comparison

The quantitative measures under the large (40 piles) scenario show that the architectural design of the cooperative framework outperforms the individual agents baseline. As shown in Table 3, the coverage of grain piles with the proposed method stands at 97.2%, a value superior to TARE (89.5%, +8.6%) and FAEL (90.8, +7.0%). The key factor contributing to this improvement is the aerial perspective: while baselines reach a “ceiling” in coverage due to occlusion on the ground, our system uses 86.8% of the additional viewpoint complementarity to fill the gap.

For statistical transparency, we report the mean and standard deviation over ten independent trials for each approach (Table 3). The proposed method delivers consistently higher grain-pile coverage and shorter completion times, while exhibiting noticeably lower variability than the baseline methods (±32 s in runtime and ±1.8% in coverage). This reduced variance suggests that the framework achieves stable, repeatable performance, rather than relying on a small number of favorable outliers.

Looking beyond the summary statistics, the results also point to a practical coverage ceiling for single-UGV exploration in this granary scenario. Both baseline approaches plateau at approximately 89−91% grain-pile coverage (Table 3), which aligns with physical visibility constraints: vertical occlusions and the limited viewing angles available from ground level restrict what the UGV can observe. As discussed in Section 2.3, this ceiling is mitigated by explicitly exploiting viewpoint complementarity. By systematically assigning grain-pile tops to the UAV and narrow aisles to the UGV, the proposed algorithm turns the aerial platform’s access advantage into improved structural coverage, thereby bypassing the inherent limitations of ground-only perception.

**Figure 6:**
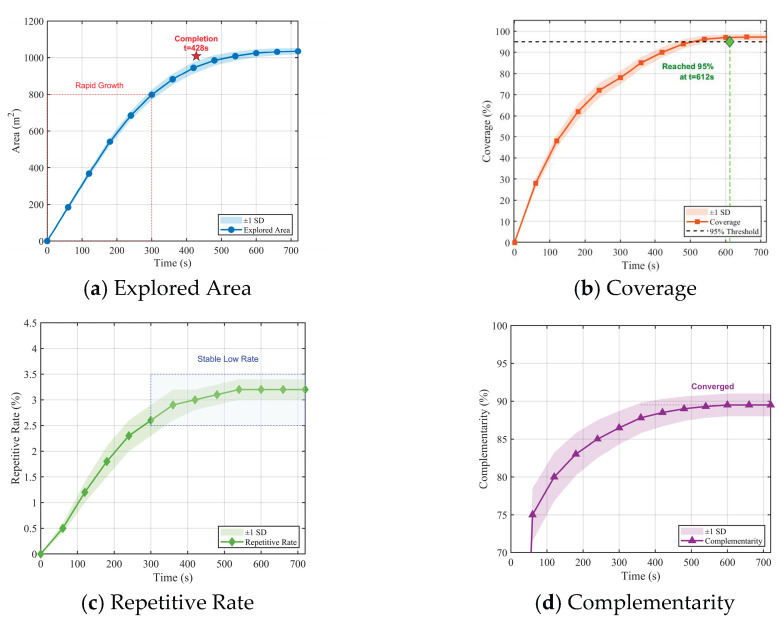
Spatiotemporal evolution of the air–ground collaborative exploration in the 40-pile scenario.

As shown in Figure 6, the spatiotemporal evolution of the air–ground collaborative exploration is illustrated. The system is first initialized, and after scanning approximately 30 grain piles, it achieves 76.0% coverage and 86.0% complementarity utilization. With a coverage of 97.2% and a comprehensive quality score of 0.91, the process concludes following global consistency checks.

#### Temporal Quality Evolution and Re-Scanning Validation

**Figure 7:**
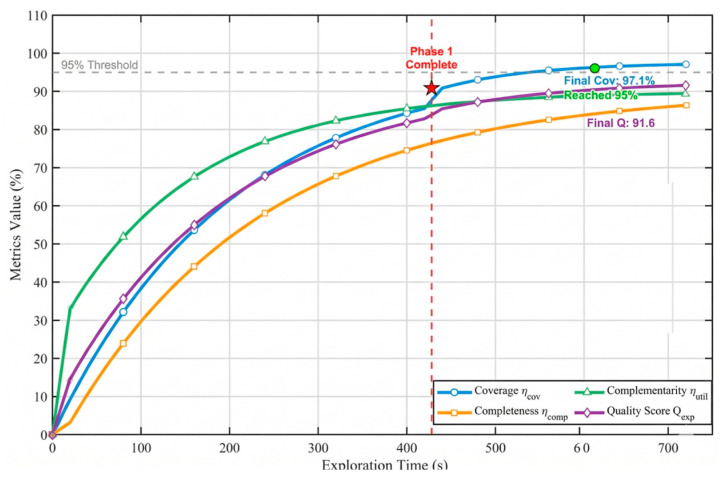
Temporal evolution of map quality metrics in the 40-pile scenario.

To assess the system’s dynamic response and evaluate the effectiveness of the closed-loop exploration strategy, the temporal evolution of quality metrics was analyzed in the 40-pile scenario, as illustrated in Figure 7.

The graph indicates that the map completeness follows a non-linear growth. It can be seen from the graph that a surge occurs at t=277.5 s (+11.8%) and t=367.5 s (+13%). These sudden upward movements correlate with the re-scanning triggers, confirming that the system effectively identified and re-scanned low-quality areas. Concurrently, the total quality score $Q_{explore}(t)$ exceeds 90% at t=245 s (see Figure 7), confirming efficient convergence. The data as a whole show that the re-scanning mechanism effectively closes the loop and prevents the premature termination of standard exploration algorithms.

### 3.4. Exploration Efficiency and Robustness Analysis

**Figure 8:**
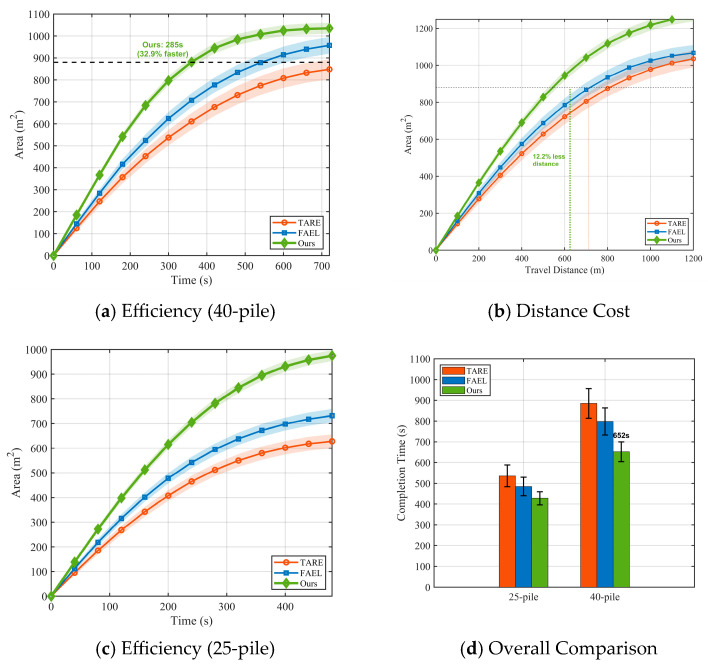
Efficiency and robustness metrics in the 25-pile and 40-pile granary scenarios.

In this section, we assess how speed and cost can be balanced. The correlation between exploration time, travel distance, and mapped area was assessed in repeated trials, with the corresponding efficiency metrics shown in Figure 8.

Noticeable divergence is shown by time efficiency (Figure 8a). The collaborative framework requires only 285 s to achieve a standard coverage benchmark of ($4\times 10^{4}m^{2}$). Based on comparison with TARE and FAEL, our method (285 s) exhibits a 32.9% acceleration over TARE (425 s) and is also considerably faster than FAEL (368 s). The system design shows the best path cost and optimization according to distance cost (Figure 8b). In addition, the total travel distance is reduced to 625 m (from 712 m for TARE), a 12.2% reduction. This efficiency stems from the viewpoint-aware allocation, which prevents the robots from driving long distances to assess obscured locations. Statistical consistency (Figure 8d), indicating robustness, shows that the proposed method achieves the smallest error margins ($\sigma_{time}$) and that reliability increases with complexity.

**Figure 9:**
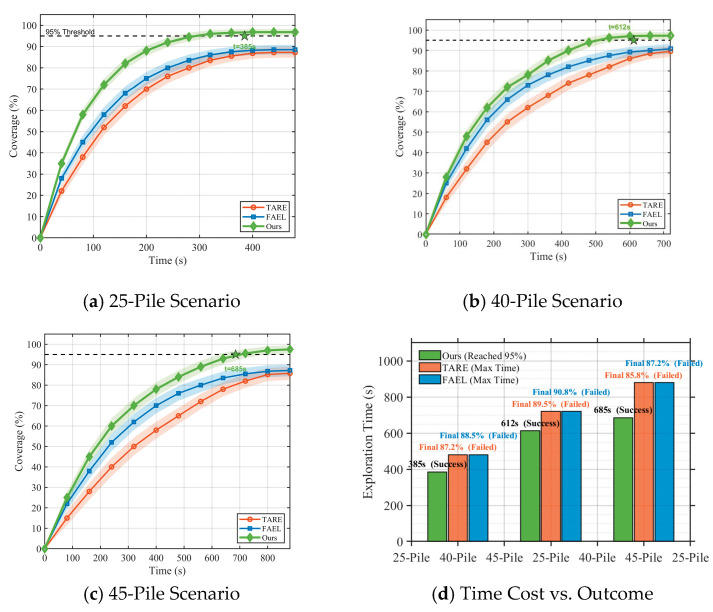
Evolution of grain pile coverage across scenario complexities.

Coverage evolution curves are shown in Figure 9. Importantly, the behavior near the end of the mission is noteworthy: TARE and FAEL plateau prematurely (never hitting 95% in any case); in comparison, the proposed method (in green) maintains the upward trend and hits the threshold.

The coverage of grain piles changes with the complexity of the scenario. Growth patterns of scenarios with 25, 40, and 45 piles are presented in a, b, and c, respectively. The proposed methodology (in green) reaches the 95% criterion significantly faster than the baselines. (d) The statistical summary reveals that TARE and FAEL failed to achieve the target limit of 95% within the time limits in all tests.

### 3.5. Scalability Test with Varying Number of Grain Piles

The results presented in Table 4 demonstrate the impact of grain pile quantity on exploration performance.

The scalability analysis provides additional support for the mechanistic explanation outlined earlier. As the number of grain piles increases from 25 to 45, the single-UGV baseline methods exhibit longer completion times and reduced coverage (Table 4). This trend points to a key limitation: in highly occluded settings, much of the additional ground motion occurs in areas that provide little new visibility, leading to diminishing returns. In contrast, the proposed approach maintains consistently high coverage (96.8–97.5%) with only a modest increase in completion time (Table 4). This robustness reflects the design of the task-allocation mechanism: by quantifying viewpoint complementarity, the framework minimizes wasted exploration effort by discouraging the UGV from targeting geometrically unfavorable regions and instead assigning those areas to the UAV (Section 2.3).

**Figure 10:**
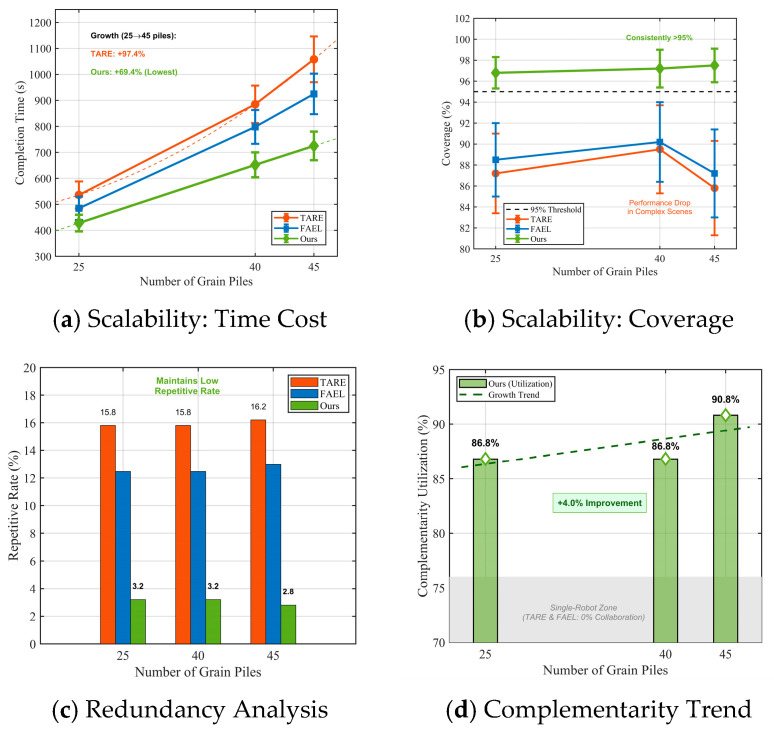
Scalability analysis under varying scene complexities.

Coverage stability stands out in our metrics. The cooperative system exhibits 96.8–97.5% coverage in the 45-pile trial; however, the baseline degrades. This resilience stems from dynamic complementarity exploitation, which, as shown in Figure 10d, increases from 86.8% in sparse maps to 90.8% in denser maps. In more complicated environments, air–ground interactions will provide more value, as our results imply.

### 3.6. Preliminary Real-World Environmental Validation

**Figure 11:**
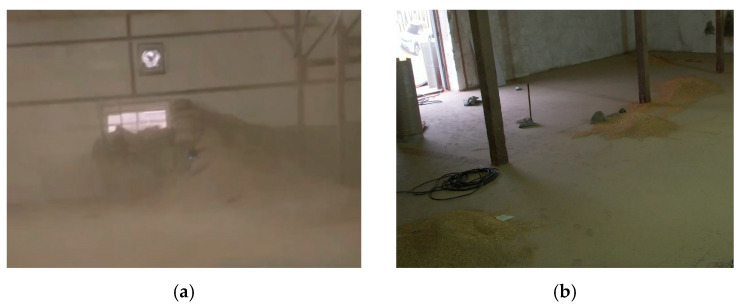
Preliminary real-world granary validation environment. (**a**) Dense airborne dust, which causes severe optical scattering and empirically justifies the distance decay factor $q_{d}$. (**b**) Local grain pile, representing the dense physical surface that the local point density factor $q_{\rho }$ aims to effectively extract from the sparse dust noise.

While the complete multi-robot collaborative exploration was evaluated on the MARSIM platform, preliminary hardware-level sensor tests were conducted in a real working granary to verify the physical assumptions underlying our dust-sensitive fusion model. Figure 11 shows the test environment, which contains high concentrations of airborne dust that visibly scatter ambient light, together with strong lighting variations.

To quantitatively evaluate the influence of the granary environment on sensor ranging performance, we conducted static benchmarking experiments in the real granary shown in Figure 11. During the tests, the ambient dust concentration was approximately 15 mg/m^3^. A high-precision laser rangefinder, with a nominal accuracy of ±1 mm, was used to provide reference ground-truth distances to a flat calibration target. Measurements from the UAV’s depth camera and the UGV’s LiDAR were collected at four preset distances: 1.0 m, 3.0 m, 5.0 m, and 8.0 m. For each distance, the Root Mean Square Error (RMSE) of each sensor was computed over 500 consecutive frames.

As shown in Table 5, the LiDAR maintained a relatively stable RMSE below 20 mm across all tested distances, indicating stronger robustness to airborne dust. By comparison, the depth camera showed a clear distance-dependent degradation trend. In particular, light scattering caused by suspended dust particles led to a sharp non-linear increase in depth-estimation error beyond 3.0 m, with the RMSE increasing from 18.4 mm at 3.0 m to 46.7 mm at 5.0 m. At 8.0 m, the depth measurements were heavily corrupted by sparse noise, resulting in an RMSE greater than 85.0 mm. These field observations provide quantitative empirical support for the introduction of the distance-decay factor $q_{d}$ in Equation (15).

In addition, the experiments showed that raw depth sensing may generate sparse floating “ghost” point clouds due to reflections from suspended dust particles. These points are typically much sparser and less spatially continuous than the point clouds returned by real grain surfaces. This observation supports the use of the local point-density factor $q_{\rho }$ to suppress airborne interference. Based on these real-world measurements, the point-wise quality model Q(p) was designed to reflect the degradation characteristics observed in actual granary environments.

Moreover, with respect to dynamic obstacles, standard industrial safety procedures in granaries generally require personnel and heavy machinery to be removed during autonomous 3D inventory mapping. Therefore, the operating environment during mapping can be reasonably treated as quasi-static, which is consistent with the assumptions adopted in our experiments.

Overall, these quantitative results indicate that, under real granary dust conditions, LiDAR maintains stable ranging accuracy, whereas the depth camera becomes increasingly unreliable at longer distances. This difference is important for the air-ground collaborative system, since it justifies assigning long-range structural perception in dusty regions with lower confidence to optical depth sensing and motivates quality-aware fusion during map construction.

## 4. Discussion

### 4.1. Efficiency Mechanism Analysis

Efficiency gains are achieved through optimal frontier allocation enabled by explicit quantification of viewpoint complementarity. By assigning UAVs to grain pile summits and restricting UGVs to aisles and lower regions, invalid motions commonly observed in homogeneous exploration are effectively avoided. In the 40-pile scenario, the proposed system completes coverage of an 8800 m^2^ area in 285 s, outperforming TARE (425 s) by 32.9% and FAEL (368 s) by 22.6%. This acceleration can be attributed to three structural factors. First, redundant exploration is largely suppressed, with the repetitive exploration rate reduced to 3.2% compared to 15.8% for TARE, saving approximately 67 s. Second, total travel distance is reduced by 12.2%, corresponding to roughly 52 s of time savings, as robots no longer move toward geometrically inaccessible targets. Lastly, the parallel air–ground workflow increases effective data acquisition throughput, enabling sensing and mapping processes to proceed concurrently.

When the reported results are critically compared with recent literature, a clear bottleneck emerges for traditional exploration. Recent confined-space studies [45] and subterranean mapping studies [46] report that single-agent systems can quickly fall into local geometric minima, resulting in redundant motion and severe backtracking. The quantitative analysis in this work further supports this trend: the single-UGV baselines (TARE and FAEL) ultimately plateau at around 89% coverage in the dense scenario, spending substantial time scanning lower aisles that are heavily occluded. This bottleneck is addressed by the proposed framework, not simply by adding a second robot, but by mathematically decoupling the operational space based on viewpoint complementarity. In particular, local minima induced by distance-based heuristics are avoided by preventing the UGV from attempting to observe regions that are effectively unobservable from ground level (i.e., pile tops).

### 4.2. Robustness and Scalability

Scalability tests display a non-linear robustness. When the complexity doubles from 25 to 45 piles, the percentage increase in the time-consuming nature of the method will amount to a mere 69.4%. This value is far lower than the linear or exponential degradation observed in the baselines. For instance, TARE exhibits a percentage increase of 97.4%. Our data show consistent standard deviations (±32–55 s), suggesting that the observed performance is not an accident but is structurally guaranteed.

This robustness is underpinned by the following:

(1) The Adaptive Utility Function is an approach in reinforcement learning that weighs gain against cost, which prevents entrapment in local optima.
(2) “Active Error Correction” ensures equality of coverage with a dedicated re-scanning loop, which is quality-driven, irrespective of pile distribution.
(3) The dust-sensitive fusion model stabilizes the sensor output of the sensor-updated map even when the dust concentration changes in the range of 5 to 15 mg/m^3^.

Densely populated areas witness an increase in the utilization of complements. One of the key strengths of this research is the fact that complex topology aids heterogeneous systems in exploring their own strengths.

Rotor downwash is a physical effect that occurs in multi-vehicle air–ground collaborative mapping in confined spaces. When the UAV flies within 3 m of a grain pile to capture surface topography, aerodynamic downwash can stir settled dust particles and generate local, dynamic dust clouds.

Although “ghost” points may appear directly in front of the image sensor and corrupt the raw 3D depth estimates, the proposed method includes a built-in mechanism to suppress this interference. In most cases, dust reflections induced by rotor downwash are unstructured, time-varying, and lack the spatial stability of solid grain surfaces.

Accordingly, the local point-density factor $q_{\rho }$ in the dust-sensitive fusion model (Equation (17)) and the incidence-angle term $q_{\theta }$ are designed to down-weight these transient, low-consistency geometric artifacts. When visual confidence drops sharply due to strong downwash, the adaptive fusion module automatically increases reliance on the ground LiDAR, which helps maintain the structural integrity of the reconstructed map.

### 4.3. Value of Re-Scanning Mechanism

Quality assurance comes at a cost. The re-scanning mechanism incurs an 8.5% time penalty (approximately 1.8 triggers per run). With this factor in mind, the Return on Investment (ROI) is significant, with enhanced coverage, completeness, and precision of reconstructed volume contained in the mesh (3D) surface.

This trade-off is non-negotiable for industrial use. In grain inventory management, an error of 4.2% corresponds to significant financial losses. The additional 8.5% task time therefore represents a negligible cost to the inventory manager, as the resulting data meet the national inventory standard, producing an overall error of less than 3%, well below the acceptable threshold.

### 4.4. Comparison with Similar Agricultural Systems

Although reminiscent of recent orchard spraying systems (e.g., Chen et al. [30]), we address a fundamentally different problem set, namely, pest biocontrol, in a distinct operational environment. Unlike orchards, which are characterized by structured, pre-determined rows of trees, granaries are unstructured, unknown environments that require interactive exploration. Consequently, our system departs from static approaches; instead of adhering to fixed formations, it utilizes real-time information gain to allocate resources dynamically. Furthermore, to ensure robust performance in harsh conditions, we introduce a ‘dust-sensitive fusion’ mechanism—a feature not typically required in outdoor agricultural robot systems.

### 4.5. Discussion on Comparability and Expected Advantages over Recent Air-Ground Systems

The performance of the proposed UAV-UGV system is quantitatively compared against that of two strong single-UGV exploration baselines. To handle the workload in heterogeneous air-ground systems, several strategies have been explored, yielding performance gains in certain cases. Beyond reporting empirical improvements, this discussion focuses on the methodology from a mechanistic perspective. This explains how the proposed method differs from recent air–ground systems, including AAGE [28] and the CERBERUS system [38,39], and why these differences matter in dusty and vertically occluded granaries.

First, task allocation in this work goes beyond distance-based heuristics. Prior air–ground frameworks often employ aerial robots to provide global coverage or to assist ground robot task assignment, typically using geometric cost, reachability, or frontier closeness as the main criteria. In granaries, however, distance is a poor proxy for observability; vertical occlusions and platform-specific viewpoint constraints largely determine what each robot can measure. In contrast, viewpoint complementarity is explicitly computed based on platform-specific information gain (Section 2.3) and is used as the primary driver for task allocation. This organizes the work roles so that UAVs are assigned to the upper parts of grain piles, while UGVs cover lower regions and narrow aisles.

Second, dust-induced measurement degradation is explicitly considered. Existing air–ground mapping pipelines are generally validated in clear-air conditions or in typical indoor/subterranean environments and do not incorporate dust-dependent confidence into fusion weights. In this work, a point-wise quality score Q(p) is assigned based on distance, incidence angle, and local point density (Section 2.4.1), and fusion weights $w_{a}$ and $w_{g}$ are adjusted dynamically according to this score. This design reduces the influence of inaccurate optical depth measurements under dusty conditions and increases reliance on LiDAR when visual depth quality degrades.

Third, quality-driven closed-loop completion is implemented. With a quality-aware re-scanning mechanism, premature termination is avoided in regions that remain low-confidence or incomplete due to uneven sensing quality. This is particularly relevant in granaries, where dust and occlusions lead to spatially non-uniform measurement reliability across the storage area.

### 4.6. Limitations and Real-World Deployment Considerations

While the proposed framework consistently outperforms existing solutions in simulation, there are several practical limitations that must be addressed prior to deployment in active granaries.

(1) UAV assignment to high altitudes requires long hover times. Thus, endurance or operational scale is an essential parameter to consider. The short flight endurance creates challenges to continuous mapping in large-scale facilities. Thus, future work should focus on endurance-aware task allocation and automatic docking.
(2) The current dust-aware fusion is calibrated for 5–15 mg/m^3^. Optical sensors may malfunction due to extreme dust during loading or unloading of grain, necessitating the addition of more robust modalities like millimetre-wave radar in the future.
(3) The simulation-to-reality gap and hardware limitations: Typical simulations assume that there is a lot of either communication or computation. In real-world settings, moving computationally intensive operations (like SLAM, map fusion) to embedded hardware may reveal bottlenecks. In addition, how algorithms speed up dreams will be challenging because of the real-world communication latency and asynchronous updates required for onboard constraints.

Future research will extend our proposed collaborative frontier estimation platform to hardware emulation and real-world experiments to reduce the gap between simulation and reality. The focus will be on tackling real-world communication delays, multi-UAV/multi-UGV swarm scaling, and dynamic obstacle avoidance.

## 5. Conclusions

Through this study, we address the significant issue of incomplete coverage in automated granary mapping. In particular, the shortcoming of single-robot systems in vertically stratified, high-dust environments. The system utilizes a “tops-for-UAV, bottoms-for-UGV” approach by placing a mathematical value on the observational advantages of aerial versus ground platforms. This logic reduces repetitive exploration by 79.7% from standard baselines. A three-factor quality model (distance, angle, density) is employed to down-weight unreliable depth data. Geometric fidelity remains largely unchanged at dust concentrations of 15 mg/m^3^. The final safety net utilizes a multi-dimensional metric to perform closed-loop re-scanning, further boosting coverage from 87% to 97.2% on autopilot. Systematic validation using the MARSIM platform confirms the effectiveness of the framework. In the complex scenario with 40 piles, the system was able to reduce the exploration time by 20.1% (428 s) with 97.2% coverage and 86.8% utilization of viewpoint complementarity. Through scalability testing, the performance remained stable despite increased environmental complexity. Ultimately, a feasible and high-precision mapping solution for intelligent granary management tasks, such as reserve estimation and pest detection, is provided. Beyond agriculture, the principles of heterogeneity-aware allocation and quality-driven active sensing may be transferable to post-disaster rescue and underground exploration, where varied robotic capabilities could be synergized. In future studies, this framework will be expanded to accommodate dynamic obstacles and scale to multi-UAV/multi-UGV swarms. Future research will extend the proposed collaborative frontier-estimation platform to hardware emulation and real-world experiments to reduce the simulation-to-reality gap. The focus will include real-world communication delays, multi-UAV/multi-UGV swarm scaling, and dynamic obstacle avoidance. Future research will extend the proposed collaborative frontier-estimation platform to hardware emulation and real-world experiments to reduce the simulation-to-reality gap, focusing on real-world communication delays, multi-UAV/multi-UGV swarm scaling, and dynamic obstacle avoidance.

## Figures and Tables

**Table 1:** Comparison of the proposed method with existing collaborative exploration frameworks.

Framework	Platform	Task Allocation Logic	Dust Degradation Handling
TARE [35]/FAEL [36]	Single UGV	Distance metrics	None
CERBERUS [37,38]	UAV + UGV (Quadruped)	Structural reachability	Standard covariance optimization
Our Method	UAV + UGV (Wheeled)	Viewpoint complementarity	Active 3-factor down-weighting

**Table 2:** Scenario parameters.

Scenario Type	Granary Size (m)	Pile Count	Pile Density (Piles/m^2^)	Pile Height Range (m)	Avg Height (m)	Dust Conc. (mg/m^3^)
Simple	50 × 20 × 7	25	0.025	1.78–3.43	2.59	5–8
Large	60 × 24 × 7	40	0.028	1.37–3.44	2.45	8–12
Dense	60 × 26 × 7	45	0.029	1.46–3.44	2.48	10–15

**Table 3:** Comprehensive Performance Comparison in Three Granary Scenarios (Mean ± Std Dev).

Algorithm	Robot Config	Completion Time (s)	Repetition Rate (%)	Pile Coverage (%)
FAEL	UGV	485 ± 45	12.5 ± 3.2	90.8 ± 3.8
TARE	UGV	536 ± 52	15.8 ± 3.5	89.5 ± 4.2
Ours	UAV + UGV	428 ± 32	3.2 ± 1.0	97.2 ± 1.8

**Table 4:** Impact of Grain Pile Quantity on Exploration Performance.

Pile Count	Our Method (s)	TARE (s)	FAEL (s)	Our Method’s Cov (%)	TARE Cov (%)	FAEL Cov (%)
25	428 ± 32	536 ± 52	485 ± 45	96.8 ± 1.5	87.2 ± 3.8	88.5 ± 3.5
40	652 ± 48	885 ± 72	798 ± 65	97.2 ± 1.8	89.5 ± 4.2	90.2 ± 3.8
45	725 ± 55	1058 ± 88	925 ± 78	97.5 ± 1.6	85.8 ± 4.5	87.2 ± 4.2
Growth Rate	69.4%	97.4%	90.7%	+0.7%	−1.6%	−1.5%

**Table 5:** RMSE of sensor depth measurements under real granary dust conditions.

Target Distance (m)	Depth Camera RMSE (mm)	LiDAR RMSE (mm)
1.0	11.5	15.2
3.0	18.4	16.1
5.0	46.7	17.5
8.0	>85.0	19.3

## Data Availability

The data presented in this study are available on request from the corresponding author.
